# Red phosphorus decorated electrospun carbon anodes for high efficiency lithium ion batteries

**DOI:** 10.1038/s41598-020-70240-6

**Published:** 2020-08-06

**Authors:** Francesco Liberale, Michele Fiore, Riccardo Ruffo, Roberto Bernasconi, Seimei Shiratori, Luca Magagnin

**Affiliations:** 1 Dipartimento di Chimica, Materiali e Ingegneria Chimica “Giulio Natta”, Politecnico di Milano, Via Mancinelli, 7, 20131 Milan, Italy; 2 Dipartimento di Scienza dei Materiali, Università degli Studi di Milano-Bicocca, Via Cozzi 55, 20125 Milan, Italy; 3 Department of Integrated Design Engineering, Faculty of Science and Technology, Keio University, 3-14-1 Hiyoshi, Kohoku-ku, Yokohama, Kanagawa 223-8522 Japan

**Keywords:** Energy storage, Batteries

## Abstract

Electrospinning is a powerful and versatile technique to produce efficient, specifically tailored and high-added value anodes for lithium ion batteries. Indeed, electrospun carbon nanofibers (CNFs) provide faster intercalation kinetics, shorter diffusion paths for ions/electrons transport and a larger number of lithium insertion sites with respect to commonly employed powder materials. With a view to further enhance battery performances, red phosphorous (RP) is considered one of the most promising materials that can be used in association with CNFs. RP/CNFs smart combinations can be exploited to overcome RP low conductivity and large volume expansion during cycling. In this context, we suggest a simple and cost effective double-step procedure to obtain high-capacity CNFs anodes and to enhance their electrochemical performances with the insertion of red phosphorous in the matrix. We propose a simple dropcasting method to confine micro- and nanosized RP particles within electrospun CNFs, thus obtaining a highly efficient, self-standing, binder-free anode. Phosphorous decorated carbon mats are characterized morphologically and tested in lithium ion batteries. Results obtained demonstrate that the reversible specific capacity and the rate capability of the obtained composite anodes is significantly improved with respect to the electrospun carbon mat alone.

## Introduction

Li-ion batteries (LIBs) represent nowadays one of the best opportunities to efficiently store chemical energy and release electric charge through electrochemical processes. Among the advantages of rechargeable Li-ion batteries over other technologies, we can cite 2–3 times higher energy densities and 5–6 times higher power densities than Ni–Cd and Ni-MH batteries^1–3^. Moreover, they have high Coulombic efficiency, low self-discharge, high operating voltage and no “memory effect”^4^. Notwithstanding, the potentialities of Li-ion batteries are still not completely exploited, mainly in some operative conditions: large polarization at high charge–discharge rates limits their power density^5^ and traditionally powder-based electrodes have long diffusion paths and slow electrode reaction kinetics^6–8^. Graphite is indeed the presently most utilized anode material for Li-ion batteries due to its low working potential, long cycle life and low cost. However, the theoretical charge capacity of the most lithium-enriched intercalation compound, LiC_6_, is of only 372 mA h g^−1^^9,10^. This paved the path towards the research of new anode materials in different, somewhere interconnecting, directions: from one side, efforts have been done to look for structures and morphologies alternative to the traditionally powder-based ones; from the other, intercalation materials different from graphite and C-derived ones have been proposed and tested.

In the contest of investigating alternative anode structures, the use of 1-D nanostructures has been proposed. With their high surface to volume ratios and reduced dimensions, they allow to shorten the longitudinal charge diffusion path and to speed up intercalation kinetics^11–18^. From the production point of view, electrospinning is an efficient, facile and versatile method to produce such 1-D carbon nanofibers (CNFs) with desired features. By tuning materials and setup parameters, the diameter and morphology of the spun fibers can be finely controlled^19–24^. Once electrospun, fibers are subjected to a carbonization process and reduced in the form of a non-woven dense carbonaceous mat, which can be used as anode in Li-ion batteries. Electrospinning-derived carbon nanofibers obtained from polyacrylonitrile (PAN) carbonization were obtained, for example, by Kim et al.^25^, by Wu et al.^26^ and by Ramakrishna et al.^27^.

The use of intercalation materials different from the carbonaceous-based ones has been proposed as well. For example, metal oxides and metal sulphides fibers were tested as anodic materials. Scientists mainly focused their attention on Li_4_Ti_5_O_12_ (LTO), TiO_2_ and TiNb_2_O_7_ as intercalation/de-intercalation metal oxides anodes materials^28–35^. Such innovative materials can also be combined with metal and carbon-based anodes. For example, the synergic positive contributions of tailored electrospun LTO and CNFs have been exploited to improve the electronic conductivity of the anode, to increase the electrolyte/electrode interface and shorten the ion transfer length^36–39^. Nam et al.^40^, by introducing Ag and Au nanoparticles into TiO_2_ electrospun fibers, demonstrated an enhanced Li-ion diffusion and charge transfer. Many other works concerning self-standing, binder- and current collector-free electrospun anodes, of carbon-based fibrous mat alone^41^ or with metals^42^, metal oxides^43–47^ and metal sulphides^48–51^ encapsulated in the electrospun CNFs are available.

Another class of materials which have been selected as potential candidates as anodes for LIBs comprises silicon^52^, germanium^53,54^, and phosphorous, which can be alloyed with Li. Phosphorus, in particular, is abundant and uniformly distributed in the earth’s crust^55–57^; moreover, being one of the main biomass constituents, can be easily recycled with no environmental concerns^55^. Black and red phosphorous, two allotropes of this element, gained attention due to their high lithium and sodium storage capacity^10,58,59^. The black allotrope, however, requires high temperatures and pressures to be processed, leaving the red one (RP) as the most promising and valuable contender to graphite as LIBs anode material^60^. Despite its high theoretical capacity of 2,596 mA h g^−1^, RP has two main drawbacks, which limit its easy and practical use: a low electronic conductivity and a ~ 300% volume expansion during lithiation and de-lithiation. This last inevitably leads to electrode pulverization, loss of contact between the active material and the current collector and therefore rapid capacity fade after the first cycle and poor electrochemical performances at high current densities. One strategy to overcome these limitations, similarly to the case of metal and metal oxides, is to create novel micro-and nano-structured carbon based anodic materials with phosphorus, mainly through ball milling and sublimation techniques^61–67^, with the dual aim of accommodating volume changes during cycling and ensuring an adequate electrical conductivity. The formation of C/P bonds allow to shorten Li^+^ diffusion path and to stabilize P during cycling and the construction of an amorphous P/C composite has been proven to guarantee the highest stability, with good rate capability and volume change accommodation during charging and discharging^2,68^. Li et al*.*^69^ used electrospinning technique to create a non-woven mat made of carbonized polymer with embedded confined RP nanoparticles, thus obtaining fast ion and electron transfer through the 3D network.

Inspired by the many recent works involving the micro- and nano tailoring of the electrode structure to speed up the intercalation kinetics and by others using high capacity materials alternative to C-based ones, we propose a simple method to obtain low-cost, environmentally friendly, highly efficient anodes for Li^+^ batteries. In particular, we decided to select one of the easiest electrospinning procedures to obtain CNFs and we tried to combine it with the use of a high capacity material, namely RP. Despite existing a high number of valid scientific publications regarding the combined synergic use of RP and C in the form of nanostructured composites, almost all the procedures require an accurate and costly handling and control on both the equipment and the materials involved. Herein we separately produced the electrospun nonwoven carbon fiber mat and consequently decorated it with RP micro-nanoparticles. PAN alone was selected as electrospun material, thus avoiding the use of coaxial elctecrospinning nozzles or the formation of complex blends. Only after polymer carbonization, the fibers network was decorated with P through a simple drop casting method in order to create a distribution of high capacity P sites in the carbon mat. This last not only acts a highly conductive path for both electrons and ions but also accommodates the volume change of P during cycling. Moreover, the porosity of the electrospun fibers facilitates electrolyte penetration and offers anchoring sites for the smaller P particles. The absence of binders, usually insulating and electrochemically inactive polymers (e.g. PVDF), allows not only to increase the volumetric and gravimetric energy density but also to reduce the possibility of side effects between the electrolyte and the binder itself, thus improving the cycling stability of the cell. Moreover, the electrode preparation process is significantly simplified, consisting only in the first basic electrospinning step and in the easy-handling red P decoration in the matrix.

## Results and discussion

### CNFs decoration procedure

The starting point for the decoration process were CNFs obtained from PAN electrospinning and subsequent carbonization. Their microstructure is well known from the existing literature, where analogous CNFs have been characterized with XRD or Raman spectroscopy. In particular, PAN fibers carbonized at 1,000 °C are characterized by an amorphous carbonaceous structure, which presents a disordered nanometric porosity^70,71^. CNFs are characterized by a high relative intensity of the Raman D band for carbon, which is indicative of a high degree of disorder in the material^70,71^.

**Figure 1:**
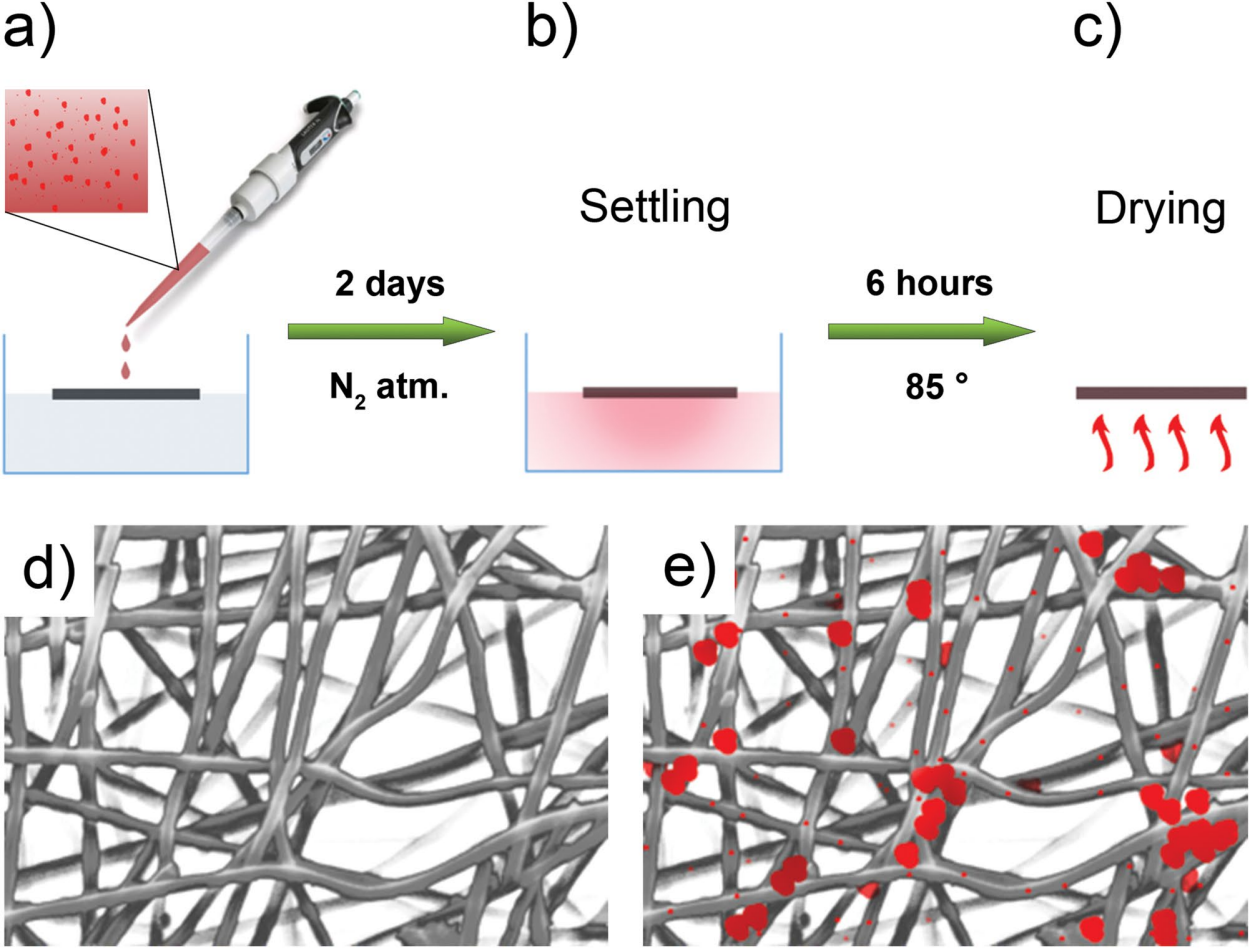
RP decorated anodes production route: dropcasting of the red phosphorous suspension (**a**), 48 h settling period (**b**), drying step (**c**); pictorial representation of the carbonaceous fibers mat before (**d**) and after (**e**) RP decoration.

Starting from CNFs, RP decorated anodes were prepared. Figure 1 shows a schematic illustration of the process for preparing the e-spun P-decorated CNFs through the drop casting of a water-based P dispersion. Initially, the RP suspension was dropcasted on the carbonized PAN mat (Fig. 1a). Subsequently, the electrode was left for 2 days in protective atmosphere (Fig. 1b). During this 48 h rest period, RP particles could penetrate in the C net, the bigger ones trapped in the first mat layers, i.e. the first few micrometers, the smaller able to penetrate deeper and anchoring to the underlying fibers. Finally, P decorated mat was dried at 85 °C (Fig. 1c). Figure 1 also schematically depicts the appearance of the mat before (Fig. 1d) and after (Fig. 1e) the decoration process.

Mat weight was measured before and after RP decoration, at the end of the drying step. The gravimetric increase of the electrodes after the RP casting step was comprised between 7 and 9% wt. for all the tested samples, being this percentage ascribable to the phosphorus added to the carbon net.

### P decorated CNFs morphological characterization

**Figure 2:**
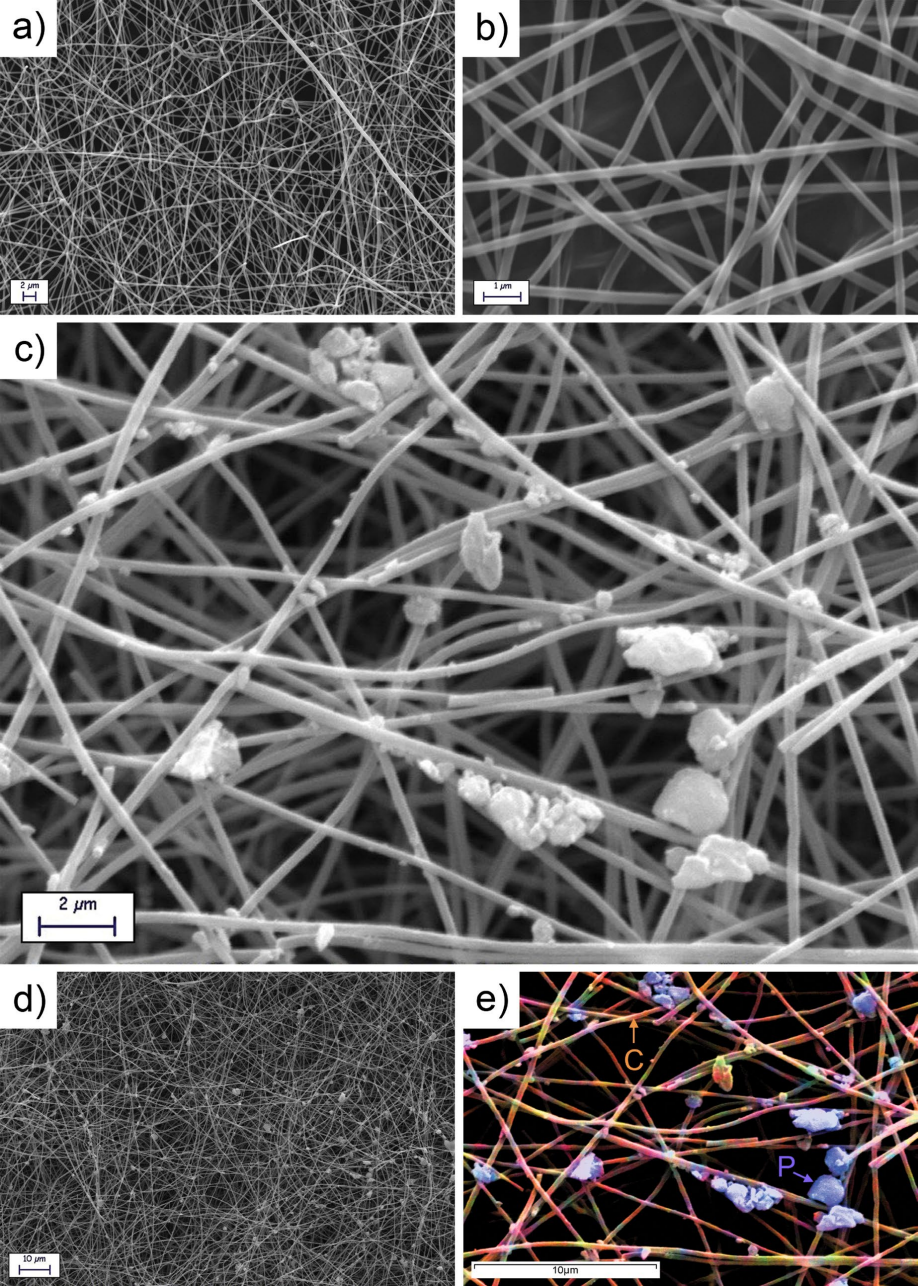
SEM micrographs of the carbonized electrospun carbon fibers (**a**, **b**) and of the same fibers after the RP drop-casting insertion process (**c**, **d**); false colors elemental EDS mapping of the fiber mat decorated with phosphorus (**e**). In the EDS mapping, carbon is highlighted in orange and phosphorus in blue.

Figure 2 shows the SEM surface morphology of the electrospun carbonized fibers alone (Fig. 2a,b) and with the incorporated RP particles (Fig. 2c–e) through the drop-casting process. The analysis was performed on the as-decorated samples, without any specific sample preparation for SEM. In both process conditions, the fibers have an average diameter of 200–300 nm, not highlighting any particular morphological difference at these magnifications.

Figure 2c and d show the fibers densely decorated with P micro- and nano-particles. In particular, it is possible to notice and assess a corroboration of our hypothesis, pictorially depicted in Fig. 1e, where the bigger particles are retained in the first few layers of the fiber mat, often in the form of aggregates, while the smaller can penetrate at deeper levels, anchoring on the underlying fibers. The considerable volume change of phosphorous during cycling, mainly of the big cluster, should not have a detrimental effect on the electrochemical performances of the battery: the breaking of the C-based fiber mat would not affect the electron and ion transport properties, resulting in a new contact between the broken fibers and other parts of the net, thus assuring the continuity of the electrode and its transport properties. To confirm the composition of our anode, differentiating the carbon matrix from the dispersed P particles, an elemental EDS mapping of the micro- and nano-sized phosphorus attached to the carbon matrix has been performed, highlighting, as shown in Fig. 2e, a uniform and widespread P distribution across the mat in addition to the one agglomerated in the form of bigger clusters at the top layers of the fibrous net.

**Figure 3:**
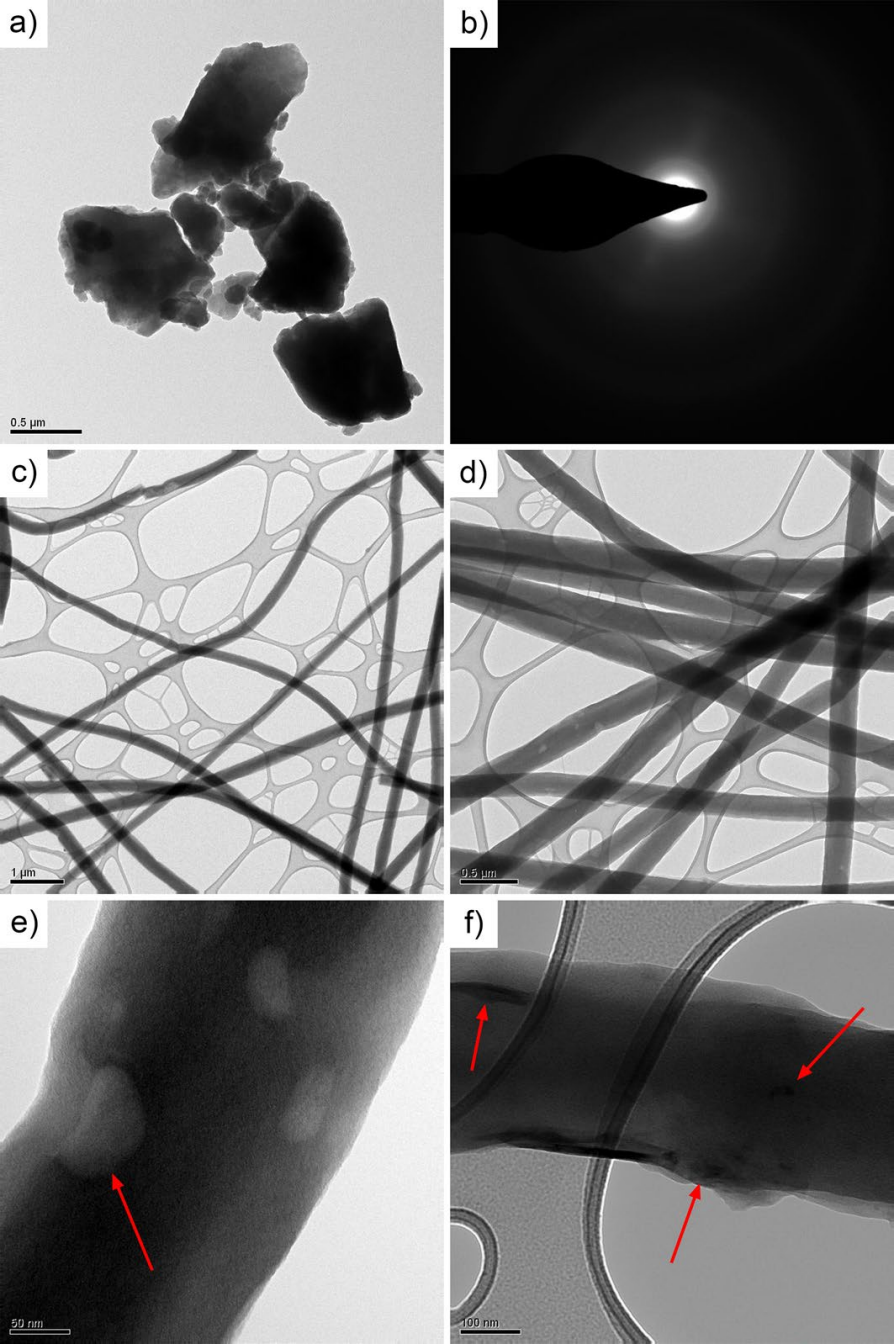
TEM of RP particles (**a**) and relative SAED pattern (**b**); TEM micrographs of the CNFs/RP composite at different magnifications (**c** and **d**); high magnification TEM image showing the surface morphology of the P-decorated fibers (**e**); detailed nanometric TEM image of the fiber´s surface aspect, with detected Phosphorus nanoclusters (**f**).

By looking at Fig. 2c, it is possible to guess the presence of very small RP particles in close contact with the fibers. Consequently, TEM was performed on the RP decorated CNFs to better investigate this aspect. The TEM analysis (Fig. 3) allowed to investigate the detailed surface morphology of the electrospun fibers, highlighting thus the possible presence of closely binded RP particles. Moreover, RP particles alone were characterized for comparison.

As visible in Fig. 3a, particles presented a broad dimensional range, ranging from few tens of nanometers to more than 700 nm. Their morphology was found to be irregular and they presented a marked tendency to agglomerate, which can support the presence of the aggregates observed in Fig. 2. The structure of RP was amorphous, as demonstrated by the selected area electron diffraction (SAED) performed on a particle (Fig. 3b). SAED also confirmed the amorphous structure of the carbonaceous fibers. Thanks to the TEM analysis performed on them, the average fiber diameter (between 200 and 400 nm) was confirmed and it was possible to observe the presence of RP nanoparticles on the surface of the fibers. It can also be noticed and appreciated how interconnected are the fibers within the mat, this guaranteeing the electrical contact and continuity also in the event of huge volume expansion during cycling. Figure 3 shows, at different magnification, the structure and morphology of the fibers, highlighting their bendability and flexibility, with the fibers overlapping in different points and thus creating preferential points for intercalation and charge-carriers exchange. During sample preparation for TEM, only some thin layers of the fibers mat were gently exfoliated and collected on the sample holder of the TEM machine. It is therefore not surprising that larger P particles are not visible, since the mechanical action required to exfoliate the layers removed them. To evaluate with higher precision the surface morphology of the fibers, the analysis was pushed to even higher magnifications and resolutions. The detailed surface aspect could be in that way studied, showing a not complete smoothness of fibers, but highlighting nanometric dimples and cavities on the external parts, as pictorially visible in Fig. 3e. The presence of this morphological disuniformities could allow an enhancement of the electrochemical active surface, thus contributing to better carrier exchange and higher specific capacities. The high resolution TEM images also allowed to confirm the presence, argued in the SEM images, of decorating P nanoparticles in close contact with the surface of the fibers and to get information about their dimensions and dispersion in the forms of small aggregates (see Fig. 3f).

### P decorated anodes electrochemical characterization

Phosphorus decorated CNFs anodes were used to assemble coin cells, which were employed for the electrochemical characterization. In general, both RP and CNFs are characterized by a great chemical stability in the electrolyte employed (LiPF_6_ in EC/DMC)^72,73^. For this reason, no uncontrolled side reactions were expected to take place during cycling.

In absence of side reactions, cells charge/discharge is based on P and C lithiation/delithiation. During the discharge step, the lithiation process occurs and there is the progressive formation of Li_n_P_m_ species (from redP to LiP_7_, Li_3_P_7_, LiP and, finally Li_3_P)^74^. Regarding carbon nanofibers, during lithiation, there is the progressive formation of Li_x_C_y_ species (from C to Li_x_C_32_, Li_x_C_18_, Li_x_C_12_, etc.). During charge, the process is reversed for both P and CNFs components.

**Figure 4:**
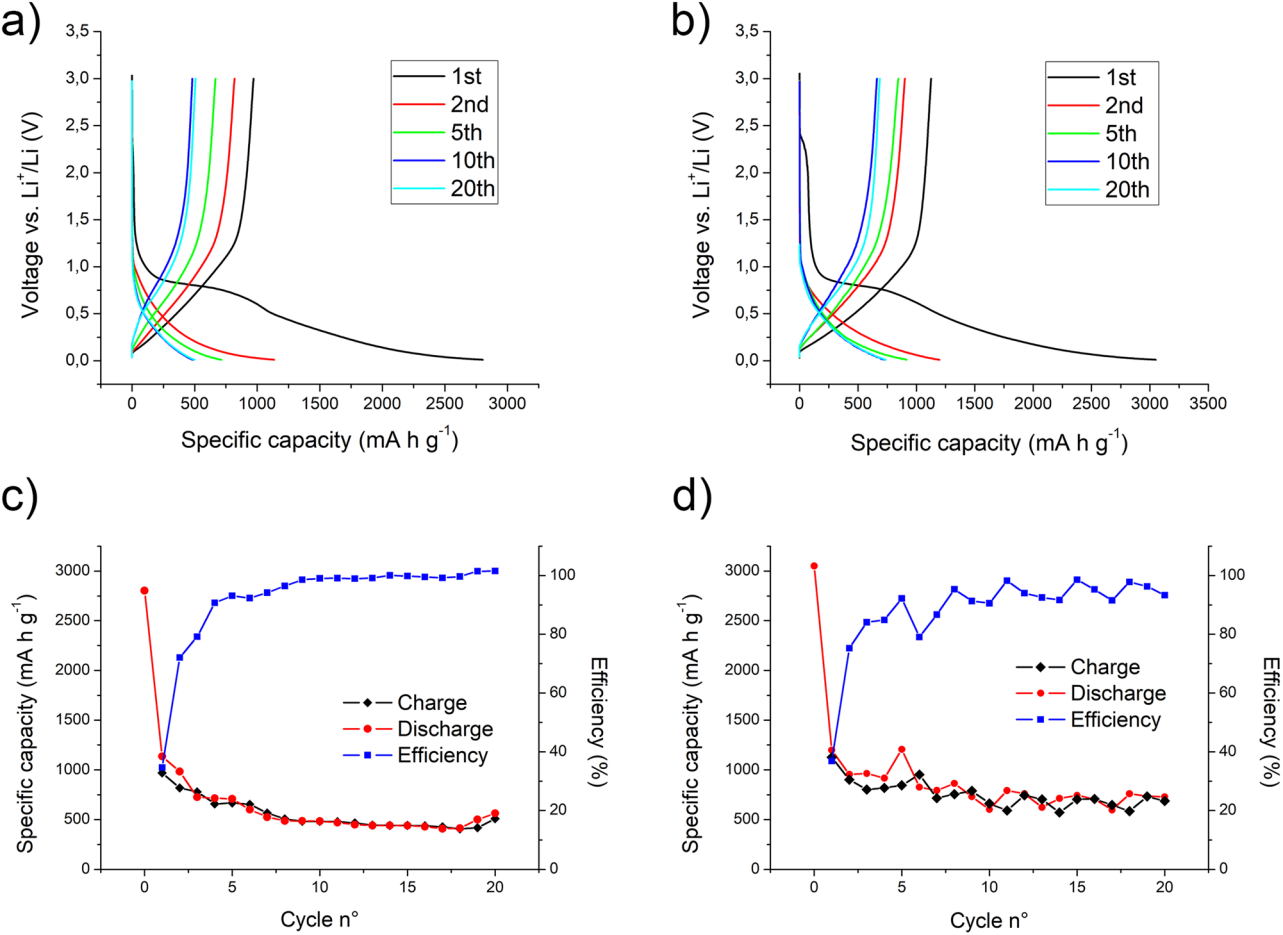
Voltage profile of CNFs (**a**) and CNFs/RP (**b**) in half cells cycled between 0.01 and 3 V versus Li+/Li at a cycling rate of ± 100 mA g^−1^. Capacity-cycle number curves of CNFs (**c**) and CNFs/RP (**d**) half cells cycled between 0.01 and 3 V versus Li+/Li at a cycling rate of ± 100 mA g^−1^, with relative efficiency.

Figure 4 collects the main electrochemical results of Li half cells tested using the electrospun carbon nanofibers as self-standing, bendable and binder-free electrodes. In particular, two different kinds of materials were tested, those made of bare electrospun PAN fibers and those with the addition of red phosphorous.

**Figure 5:**
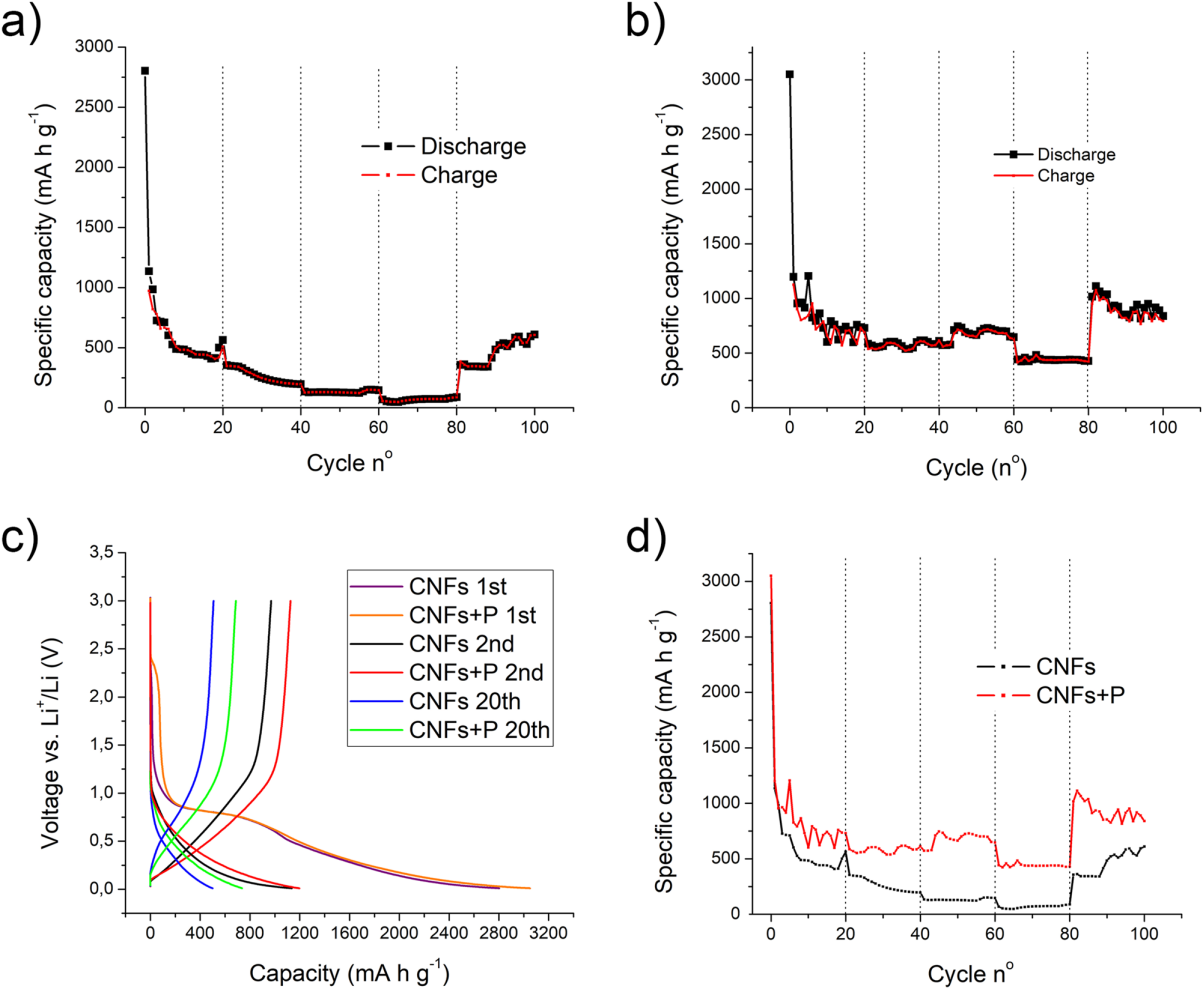
Capacity of CNFs (**a**) and CNFs/RP (**b**) LIBs as a function of the discharge rate (100, 200, 300, 600, 100 mA g^−1^). (**c**) comparison of the voltage profiles of CNFs and CNFs/RP LIBs cycled between 0.01 and 3 V versus Li+/Li at a cycling rate of ± 100 mA g^−1^ (1st, 2nd, 20th cycles). (**d**) comparison of capacities of CNFs and CNFs/RP LIBs as a function of the discharge rate (100, 200, 300, 600, 100 mA g^−1^).

Figure 4a shows the first, second, fifth, tenth and twentieth charge and discharge cycles for the CNFs’ based-battery. It can be observed a high initial cathodic capacity, reaching a value higher than 2,800 mA h g^−1^ when the lower voltage limit of 10 mV was achieved imposing a current of − 100 mA g^−1^. A fast capacity drop is detected after the first discharge (anodic branch), reasonably due to the irreversible reactions at the anode/electrolyte interface leading to the solid-electrolyte interface (SEI) formation. From the second to the tenth cycle, the usual constant capacity decay, both in the charge and discharge branches, is observed, as more clearly pictorially depicted in Fig. 4c: the specific capacity passes from the outstanding values of 1,136 mA h g^−1^ for the second discharge and of 970 mA h g^−1^ for the first charge to values, respectively, of 486 mA h g^−1^ and of 482 mA h g^−1^ for the tenth discharge and charge cycles. This behavior was due to the completion, within the first stages, of the irreversible capacity-consumption side reactions at the electrode, inside the electrolyte and between the two. After that, the discharge and charge values does not change significantly, but, reversely, there is a mild capacity recovery in both charge and discharge profiles from the 10th to the 20th cycle, as observable comparing the blue and cyan lines in Fig. 4a and the values reported in Fig. 4c. Being this study focused on the application of carbon based negative electrodes, the charge efficiency is computed as the anodic charge on the cathodic one. As can be observed in Fig. 4c, efficiency values are higher than 90% after the first three cycles and approach 100% during the remaining part of the test. The same tests, with imposed currents of ± 100 mA g^−1^ within the 3–0.01 V range, were afterward conducted for the phosphorous-decorated fibers, and the results are reported in Fig. 4b and d. The profile of Fig. 4b almost exactly replicate that of Fig. 4a and the same consideration previously reported are applicable both for the first cycle capacity drop and the stabilization trend after the tenth cycle. A noteworthy consideration is an overall shift towards higher capacity values for all the cycles in both positive and negative branches, as more clearly observable in Fig. 5a, where the first, second and twentieth cycle of the two kinds of batteries are depicted and compared. Moreover, a slightly more oscillating variation of the charge and discharge values are detected, thus leading to a less stable efficiency profile in Fig. 4d. The presence of a complex phosphorous distribution inside the fibrous mat could cause the selective activation of intercalation sites within the material during different cycles: the access of the electrolyte and its efficient contact with the P nanoparticles embedded within the dense and complex net could differ significantly cycle to cycle, not guarantying a perfectly constant contribution of P to the performances’ improvements. The oscillation in the Coulombic efficiency can be indeed ascribed to a not perfect distribution of the particles across the electrode section. The high capacities obtained upon cycling are an indication of the stability of the CNFs/RP composite, with no macroscopic evidence of degradation within the voltage scan range.

The rate capability of the electrode material is of fundamental importance when evaluating the practical application in the device. Figure 5a and b show the rate performance of both CNFs and CNFs/RP materials for LIBs. In both cases the current density was increased each 20 cycles, starting from 100 mA g^−1^, passing to 200, 300, 600 mA g^−1^ and then back again to 100 mA g^−1^.

The pure CNFs electrode (Fig. 5a) displayed capacities around 410, 200, 150 and 85 mA h g^−1^ at the end of each of the first four sequences, showing a rapid recovery to 356 mA h g^−1^ at the beginning of the last sequence, i.e. when the initial current density of 100 mA g^−1^ was restored. Afterwards, a continuous constant rise till the last capacity value of 600 mA h g^−1^ was recorded. The fact that the capacity shows a slight increase in the last 20 cycles compared to the first 20 ones at the same charge/discharge currents can be accounted to the need of the material of a sort of “stabilization time” for displaying all its potentiality: being the electrode made of densely intertwined fibers, it could require several charge/discharge cycles to make all the carbon fibers accessible to the electrolyte and, therefore, electrochemically active and make the overall electron- and ion-transport effective and efficient. The same cycles sequence, with identical charge and discharge current densities, was performed using the CNFs/RP composite. Specific capacity values of 730, 584, 645 and 429 mA h g^−1^ were recorded at the end of the first, second, third and fourth sequence, respectively. In the first 20 cycles, a quite important oscillation of the charge/discharge capacity values was detected, these again due to the not immediate, prompt and uniform activation of all the intercalation sites in the P-decorated mat within the first stages. Passing to the second sequence, with the current density doubled from 100 to 200 mA g^−1^, as expected, a decreasing step in the capacity is observed. What is counterintuitive and quite misleading is the capacity rise recorded in the third sequence, the one with an imposed ± 300 mA g^−1^ current density. This behavior can be explained with the progressively increasing growth of the number of P active alloying elements within the carbon matrix: not only time, but also higher current values can be therefore needed to overcome the P/carbon and P/electrolyte interface potential barrier, guarantee the electron- and ion-exchange, and make the intercalation completely feasible. When a current 6 times higher with respect to the initial one was imposed for other 20 cycles, i.e. ± 600 mA g^−1^, a slight capacity drop was registered and the corresponding capacities ranged between 422 and 482 mA h g^−1^, anyway high outstanding values at these current densities. Reversing again the current densities to ± 100 mA g^−1^, the capacity increased to more than 1,000 mA h g^−1^, settling to values of around 800 mA h g^−1^ at the end of the test, confirming the capacity recovery trend over tens of cycles after the first stages of the battery life. Figure 5c shows a comparison between the first, second and twentieth charge and discharge cycles for CNFs and CNFs/RP based-batteries. It is evident, from this direct comparison, that CNFs/RP anodes resulted in systematically higher capacities with respect to CNFs alone. By comparing the rate capability of LIBs made with CNFs and CNFs/RP electrodes (Fig. 5d), it is clearly observable the positive synergic effect of the CNFs/RP matrix, which allows to get a significant increase in the LIB’s specific capacity with respect to the CNFs alone, despite presenting an overall less stable profile.

## Conclusions

With this study, it was possible to show an easy-handling and efficient process to decorate self-produced electrospun carbon fibers with red phosphorus. The proposed drop casting technique turns out to be as efficient as other CNFs/RP decoration processes, with the great advantage of exploiting a simple and cost-effective procedure. The obtained composite compound was used as anode for Li-ion battery, showing relevant electrochemical performances in half-cell, greatly enhanced with respect to the bare carbon fibers. SEM and TEM microscopic techniques allowed to assess the widespread distribution of the RP particles within the fiber mat. The electrochemical evidences showed the stability of the system also at severe charge and discharge rate conditions. A further optimization of the RP dispersion phase before the casting step would allow to obtain an even more uniform distribution of the particles within the fiber mat, resulting in an improved stability of the electrode upon cycling. Moreover, the observed oscillation in the Coulombic efficiency resulting from the not perfect distribution of the particles across the electrode section can be overcome by performing a double-step sequential decoration process from both the CNFs electrode’s surfaces.

## Methods

### Materials and chemicals

Polyacrylonitrile (PAN, average *Mw* ~ 150,000) was purchased from Sigma-Aldrich (USA). N, N dimethylformamide (DMF; 99.5%) was purchased from Tokyo Chemical Industry Co. Inc. (Tokyo, Japan). Red Phosphorous (RP, purity ≥ 97%) was purchased from Sigma-Aldrich (Italy). Plastic syringes and needles (21G 1/2) were purchased from Terumo (Tokyo, Japan). LiPF_6_ (1 mol L^−1^) in ethylene carbonate (EC)/dimethyl carbonate (DMC) (1:1 v/v%) solution was purchased from Sigma-Aldrich (Italy). Cr2032 coin cells components were purchased from MTI Corporation, KJ Group (USA).

### CNFs electrospinning and carbonization

A methodology already reported in literature was employed to produce CNFs^75^. Before electrospinning, PAN (10 wt%) was dissolved in DMF and stirred for at least 48 h at 60 °C. The resulting solution was loaded into a plastic syringe, which was mounted on the electrospinning setup. Planar substrates, made of aluminum, were mounted on a metal collector. During electrospinning, the voltage applied was set to 10 kV, the distance between the needle tip and the collector was set to 15 cm, the solution flow rate was fixed at 1.0 mL h^−1^ and relative humidity was maintained around 30–40% by employing silica gel. After electrospinning, as-spun membranes were removed from the Al substrates and cut into 3 cm × 3 cm pieces. The membranes were then sandwiched between flat metal plates covered by a PTFE film and placed in a hot press set at zero pressure until the temperature of the plates reached 110 °C. Then, 2 MPa pressure was applied for 3 min. Subsequently, pressure was released completely and the sample was allowed to cool down to room temperature. During carbonization, the PAN nanofibers were first stabilized in air at 280 °C for 2 h (heating rate: 1 °C min^−1^) and then carbonized in N_2_ atmosphere at 1,000 °C for 1 h (heating rate: 5 °C min^−1^).

### P decorated anodes preparation

The self-standing CNFs mat was placed in a beaker filled with water over a soft spongy polystyrene base: manually grinded RP dispersed in water (1 g/l) was gently drop-cast on the CNFs layer, ensuring that its entire surface was covered by the RP dispersion. The RP impregnated carbon mat was left 2 days in dry-box under N_2_ atmosphere and then dried at 85 °C for 6 h, letting all the absorbed water evaporate.

### Anodes electrochemical characterization

The electrochemical properties of the self-standing carbon/RP composite nanofibers were characterized using 2032 coin cells in an half cell configuration with metallic lithium as counter electrode. Neither metallic current collector nor polyvinylidene fluoride (PVDF) binder were used in the preparation of the working electrodes for these 2032 coin cells. The separator consisted of a 25 μm microporous monolayer membrane (Celgard 2500, Celgard, Charlotte, North Carolina, USA) impregnated with 12 drops of electrolyte (1 mol L^−1^ LiPF_6_ in EC/DMC—1:1 v/v%). The cells were assembled in a dry air-filled glove box (Galaxy, Matsuura Manufactory Corporation, Tokyo, Japan) and cycled in the voltage range of 3.0–0.01 V with a multichannel charge–discharge device (HJ-1001SMB, Hokuto Denko Corporation, Tokyo, Japan). The current value for the 1st to 20th charge–discharge cycles was 100 mA g^−1^ for both the C and the C/P anode fibrous matrix. The current was then increased to 200, 300 and 600 mA g^−1^ every twenty cycles for testing the capacity variation at different C rates. Finally, the current value was reverted to the initial value of 100 mA g^−1^ for other 20 cycles to investigate the capacity retention.

### Morphological characterization

The surface morphology of the self-standing carbon nanofibers mat was determined with a field emission scanning electron microscope (Zeiss Evo 50 EP) equipped with EDS module (Oxford Inca Energy 200) for the elemental analysis of the P-enriched carbon fibers. Samples were directly analyzed, without any specific preparation for SEM. For the detailed morphological microstructure characterization, a transmission electron microscope (Philips CM 200) was adopted. Samples were prepared for TEM by gently exfoliating and collecting on the sample holder of the TEM machine only some thin layers of the fibers mats.

## Data Availability

The datasets generated during and/or analyzed during the current study are available from the corresponding author on reasonable request.
